# Transfusion of Blood

**Published:** 1889-10-12

**Authors:** 


### RECENT RESEARCH.

### TRANSFUSION OF BLOOD.

Dr. William Hunter, John Lucas Walker Student in Pathology, Cambridge, has recently delivered three lectures at the Royal College of Surgeons on transfusion of blood, from which the following conclusions may be drawn. Transfused blood possesses no nutritive value. It is therefore useless in all cases of atrophic anæmia, by which term is signified the anæmia of wasting diseases. But if the condition to be met is a want of red blood corpuscles, then transfusion may possibly be of value. Transfusion of blood may also possibly be of value in carbonic oxide poisoning, where the red corpuscles are incapacitated for their work by a close union of the gas with the hæmoglobin of the corpuscles. Transfusion, again, is not indicated in anæmia of idiopathic or traumatic origin. Examples of idiopathic anæmia are chlorosis lencocythæmia and pernicious anæmia, in all of which transfusion is more or less useless. As regards traumatic anæmia, subsequent recovery, after great losses of blood and transfusion in animals, has not been accelerated. Lastly, if the condition to be met is a threatened failure of circulation, as the result of sudden loss of blood, then it is unnecessary to have recourse to blood transfusion, as the infusion of a neutral salt meets equally well, if not better, all the indications. For practical purposes the lecturer states, as the result of his experiments, that all the advantages of blood transfusion may be more readily obtained by the infusion of a neutral saline, such as a ¾ per cent. of a solution of common salt (about one drachm to the pint). The only pressing indication for the performance of transfusion is the collapse from sudden and severe losses of blood. But the value possessed by transfused blood in such cases is almost solely in virtue of its physical properties. The chief physical property of blood for purposes of transfusion is undoubtedly its volume. From sudden loss of blood the pressure falls rapidly, and death will ensue unless means be taken to meet the sudden failure of the circulation. The readiest way in which this can be done is to replace the lost blood by a certain quantity of fluid. In an emergency, the infusion of ordinary water has been followed by results as successful as any obtained after transfusion of blood. But transfusion of either blood or of any other fluid must be extremely limited in its application, even to those comparatively few cases in which it is indicated, for certain instruments are required which are not always at hand on an emergency, and certain precautions are necessary which may not occur to others than experimental experts.

